# Comparative Genomic Analysis of *Sphingomonas morindae* sp. NBD5 and *Sphingopyxis* sp. USTB-05 for Producing Macular Pigment

**DOI:** 10.3390/microorganisms11020266

**Published:** 2023-01-19

**Authors:** Chao Liu, Zhenzhen Zhao, Qianqian Xu, Haiyang Zhang, Xiaolu Liu, Chunhua Yin, Hai Yan, Yang Liu

**Affiliations:** School of Chemistry and Biological Engineering, University of Science and Technology Beijing, Beijing 100083, China

**Keywords:** Sphingomonadaceae, macular pigment, bacterial biosynthesis, genome analysis

## Abstract

*Sphingomonas morindae* sp. NBD5, which we previously identified and tested, is a new bacterial strain for producing lutein. Here, based on the next-generation sequencing technology, we analyzed high throughput genomic sequences and compared related functional genes of *Sphingomonas morindae* sp. NBD5 and *Sphingopyxis* sp. USTB-05. The genome of *Sphingomonas morindae* sp. NBD5 has two sets of chromosomes, which is 4,239,716 bp and harbors 3882 protein coding genes. There are 59 protein-coding genes related to the macular pigment (MP) biosynthesis, of which four genes (*ackA*, *pgm*, *gpmI* and *pckA*) are unique. These genes, *pckG*, *porB*, *meh*, and *fldA*, are unique in *Sphingopyxis* sp. USTB-05. The analysis of *Sphingomonas morindae* sp. NBD5 and *Sphingopyxis* sp. USTB-05 genomes gives an insight into the new pathway for MP production. These genes for the transformation of glucose to MP were also found in *Sphingomonas morindae* sp. NBD5 and *Sphingopyxis* sp. USTB-05. This study expands the understanding of the pathway for complete biosynthesis of MP by *Sphingomonas morindae* sp. NBD5 and *Sphingopyxis* sp. USTB-05.

## 1. Introduction

Macular pigment (MP) is found in the macular region of the primate retina, and consists mainly of lutein and zeaxanthin that cannot be synthesized by the body itself [1,2]. Furthermore, the detection of lutein and zeaxanthin in the macular region of the retina can be used as the optical density of macular pigment (MPOD), which can be used as a biomarker to predict ophthalmic diseases and visual function [3]. Cataracts and age-related macular degeneration (AMD) are the leading causes of visual impairment and acquired blindness, affecting tens of millions of people worldwide to varying degrees. Although MP must be obtained from daily food, most people’s daily intake is seriously insufficient. The latest research shows that daily intake of 10 mg lutein and 2 mg zeaxanthin can improve visual function and delay the development of AMD [4,5]. Lutein can be provided to fetuses and infants through umbilical cord blood, breast milk, and diet. Lutein can accumulate in human oxidative stress and high metabolic organs, indicating that it plays a unique role in the development of human embryonic organs and the development of infant eyes and brain [6]. By investigating the changes in carotenoid content of pregnant women throughout pregnancy, it was determined that prenatal maternal lutein and zeaxanthin supplementation could offset maternal carotenoid consumption and improve maternal and infant physical fitness to a certain extent [7].

Lutein and zeaxanthin are two of the 600 carotenoids in nature, which are deposited in the retina (macula) of the eye. They can be biosynthesis, which widely exists in egg yolk, corn, vegetables and fruits [8]. At present, there are many reports on eukaryotic biosynthesis of carotenoids. Their synthesis pathway has been clarified in orange-yellow fruits such as marigold flowers, mangoes, broccoli, watercress, and other green vegetables [9,10,11]. As far as production is concerned, the cultivation of marigold is a means of obtaining natural lutein and lutein esters. As far as microorganisms are concerned, free lutein can be biosynthesized by microalgae [9]. However, the production of lutein and zeaxanthin by prokaryotes was reported rarely.

*Sphingomonas morindae* sp. NBD5 was a new species that was identified as this genus. Phylogenetic analysis showed that it belonged to the Sphingomonadaceae family [12]. In our previous research, *Sphingomonas morindae* sp. NBD5 had the function of producing lutein [13]. *Sphingopyxis* sp. USTB-05 is a well-known strain that can be used to completely biodegrade cyanobacterium hepatotoxin [14], but whether it can produce lutein has not been investigated. Genomic studies of MP producing bacterial strains are rarely reported, and numerous genes encoding MP synthases need to be elucidated. Therefore, in order to fully elucidate the MP biosynthesis pathway in *Sphingomonas morindae* sp. NBD5 and *Sphingopyxis* sp. USTB-05, it is very important to discover the encoding genes and enzymes through genome mining. Here, we firstly sequenced the genome of *Sphingomonas morindae* sp. NBD5, which was 4,239,716 bp and contained 3882 protein coding genes. Glucose converted to lutein or zeaxanthin pathways were discovered in *Sphingomonas morindae* sp. NBD5 and *Sphingopyxis* sp. USTB-05.

## 2. Materials and Methods

### 2.1. Bacterial Strains

*Sphingomonas morindae* sp. NBD5 was isolated and identified from Noni (*Morinda citrifolia* L.) branch [12]. *Sphingopyxis* sp. USTB-05, which we previously sequenced, was isolated and identified from the sediment of Dianchi Lake in Kunming, Yunnan, China [15]. Strain NBD5 and USTB-05 were grown in LB media (3.3 g/L peptone, 1.7 g/L yeast extract, 3.3 g/L NaCl), and incubated aerobically at 30 °C.

### 2.2. DNA Extraction and Sequencing

The colonies were obtained after 48 h of incubation on LB agar medium at 30 °C. A single colony was picked and cultured for 48 h at 30 °C, 200 rpm to logarithmic phase in LB liquid media. Cells were collected by centrifugation at 13,282× *g* for 10 min at 4 °C, and the supernatant was discarded. Genomic DNA was extracted using the Rapid Bacterial Genomic DNA Isolation Kit (CoWin Biosciences, Taizhou, Jiangsu, China) according to the manufacturer’s instructions. DNA quality, concentration and integrity were checked by NanoDrop (Thermo Fisher Scientific, Waltham, MA, USA) and gel electrophoresis. DNA damage repair and end repair, magnetic beads purification and linker connection were processed by using the official SQK-LSK ligation kit (BAIYITECH, Hangzhou, Zhejiang, China). The third-generation genomic library was constructed by the standard protocol of Oxford Nanopore Technologies (ONT, Oxford, UK). High quality data sets with corresponding sequencing depths of 100-fold were generated (Figure S1). Large fragments of DNA were recovered by using BluePippin (Notre Dame, IN, USA) automatic nucleic acid recovery system [16]. The second-generation genomic library of strain NBD5 was performed using the Illumina X10 platform (Madison, WI, USA) [17].

### 2.3. Genome Assembly and Quality Control

The subreads used second-generation sequencing technology were filtered by fastp software v0.23.2. These sequences with a quality value of Q < 25 were deleted. The original fastq format was obtained by base calling FAST5 file through Albacore software in MinKNOW software v4.0.4. The complete map of the strain NBD5 genome was assembled using SPAdes software v3.13.0. Meanwhile, Pilon software v1.5 was used to correct the assembly results.

### 2.4. Genome Annotation

The online NMPDR-rust server was used to predict coding sequences (CDSs) region of the assembly sequence. tRNA were predicted using tRNAscan-SE v2.0. All unigenes were functionally annotated using the Swiss-Prot and Pfam databases. Circos software was used to integrate the COG annotation results, methylation results, RNA annotation results, GC content, and GC-skew to map the entire genome of the bacterial strain. Function annotation of NBD5 and USTB-05 were searched for Kyoto Encyclopedia of Genes and Genomes (KEGG), Cluster of Orthologous Group (COG), Gene ontology (GO) to predict metabolic information using BLAST software v2.9.0 [18]. In addition, CRISPRFinder software v4.2.19 was used to predict the clustered regularly interspaced short palindromic repeats (CRISPR) structure of the strain NBD5 genome [19]. The coding sequences of the genome were aligned using MUMmer version 4.0+ and analyzed in conjunction with the results of the genome annotation [20].

### 2.5. Detection of Lutein of Sphingomonas morindae sp. NBD5

The crude extract of *Sphingomonas morindae* sp. NBD5 was obtained by 0.22 um organic filter membrane filtration. The crude extract of *Sphingomonas morindae* sp. NBD5 and standard lutein were detected on a Pillar Agilent Infinity Lab Poroshell 120 SB-C_18_ column (2.7 µm, 100 × 3.0 mm i.d.) (Agilent Technologies Inc., USA) in a liquid chromatography-mass spectrometry quadrupole time of flight (LC-Q-TOF/MS) (Agilent Technologies Inc., USA), connected to a UV-visible absorption spectrum detector. The crude extract of *Sphingomonas morindae* sp. NBD5 and standard lutein were dissolved in chemical reagent (ethanol/n-hexane, 1:6.7, *v/v*). The column was kept at 30 °C. The mobile phase was a mixed solution of methanol/water (97.5:2.5, *v/v*) at a flow rate of 1 mL/min. The injection volume was 5 µL. The MS parameters were set as follows: splitting voltage of 4000 V, collision energy of 15 eV, dry gas (N_2_) temperature of 325 °C at 10 L/min. MS spectra were acquired in the range of *m/z* 50 to 700 and MS/MS was obtained in automatic mode.

### 2.6. Data and Drawing Tool

The genome circular map was generated by using the online software Circos (http://www.circos.ca, accessed on 4 June 2022). The heat map was generated by using the online software Hiplot (https://hiplot.com.cn, accessed on 1 May 2021). The sequence data were submitted to NCBI Sequence Read Archive (https://www.ncbi.nlm.nih.gov/sra/, accessed on 8 March 2020) with accession numbers CP084712, CP084930-CP084933. The sequence data will be released on 31 October 2023.

## 3. Results

### 3.1. General Features of the Genome

**Figure 1:**
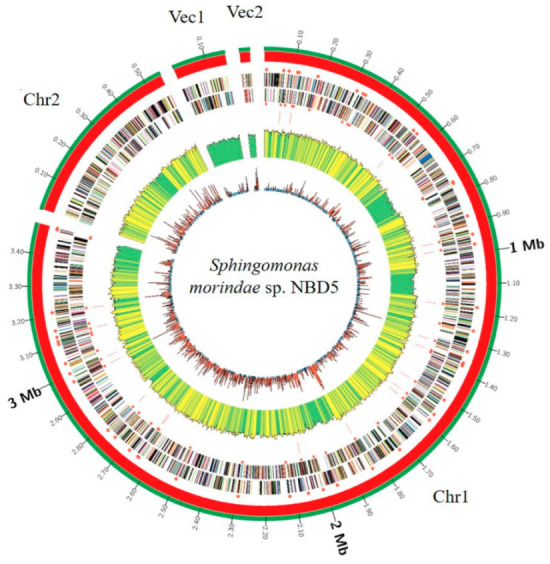
Circular representation of two chromosomes and two plasmids of *Sphingomonas morindae* sp. NBD5. Marked information is illustrated from the inner to outer ring: GC skew, GC content, the distribution of rRNAs and tRNAs, CDSs on the reverse stand, CDSs on the forward stand, genomes size.

*Sphingomonas morindae* sp. NBD5 contains two chromosomes and two plasmids (Figure 1). The genome of 4,239,716 bases is, respectively, predicted guanine-cytosine (GC) content of 70%, 61 tRNA genes, 1 tmRNA gene and 3882 protein coding sequences (CDSs), accounting for 62.39% of the total coding sequences. The 16S rRNA of strain NBD5 has three complete copies (Table 1).

### 3.2. Gene Ontology Annotation

**Figure 2:**
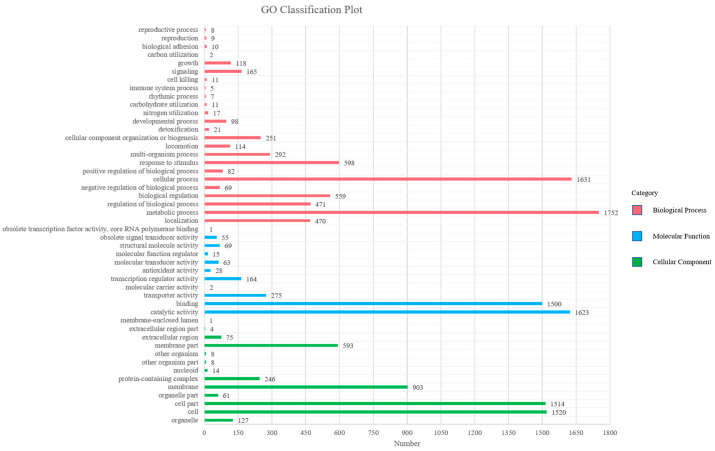
Distributions of second level GO of *Sphingomonas morindae* sp. NBD5 genome sequence. The *y*-axis indicates the GO ontology; the *x*-axis represents the number of unigenes in a category.

Gene ontology (GO) is a standardized gene functional classification system, which tenders a dynamic-updated controlled vocabulary. The GO analysis indicated that a total of 15,640 GO terms were associated with all unigenes (Figure 2, Supplementary Table S1). According to the secondary classification of the GO terms, all unigenes are sorted into 48 functional groups. Biological process is the main category of GO annotations (6771, 43.29%) unigenes, followed by cellular component (5074, 32.44%) and molecular function (3795, 24.26%). The metabolic process (25.88%) and cellular process (24.09%) represent most of the biological process category, indicating that the bacterium has high metabolic activity. There are also some subcategories including response to stimulus (8.83%), biological regulation (8.26%), regulation of biological process (6.96%), localization (6.94%), multi-organism process (4.31%) and cellular component organization or biogenesis (3.71%). Cell (29.96%), cell part (29.84%), membrane (17.80%), membrane part (11.69%) and protein containing complex (4.85%) are the cell gene clustering of five main components. The catalytic activity (42.77%) and binding (39.53%) represent most of the molecular function category, forecasting that the bacterium has a high degree of molecular catalysis (Figure 2).

### 3.3. Cluster of Orthologous Groups Annotation

**Figure 3:**
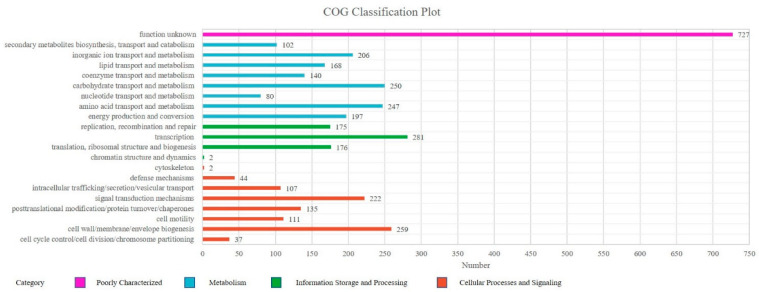
COG functional classification of *Sphingomonas morindae* sp. NBD5. The columns represent the number of unigenes in each subcategory.

Cluster of Orthologous Groups (COG) is a database for homologous classification of gene products. A total of 3668 classified unigenes were divided into 21 functional categories. Analysis of the assembled *Sphingomonas morindae* sp. NBD5 unigenes showed that 80.18% of unigenes were annotated in the COG database. Among these categories, the five main groups of transcription (281, 7.66%); cell wall, membrane, envelope biogenesis (259, 7.06%); carbohydrate transport and metabolism (250, 6.82%); amino acid transport and metabolism (247, 6.73%) and function unknown (727, 19.82%) are the most prevalent. The biosynthesis of lutein is completed by carbohydrate transport and metabolism (6.82%), this is also a significant group to be considered. Additionally, the biosynthesis of lutein depends on various biological enzymes, which are synthesized by cellular processes and signaling (25.00%) and metabolism (37.90%). Thus, posttranslational modification, protein turnover, chaperones (3.68%) is also considered an important functional group (Figure 3, Supplementary Table S2).

### 3.4. Gene Clusters and Pathway Associated with Lutein and Zeaxanthin Biosynthesis

Lutein and zeaxanthin contain two ionone rings in its chemical formula. They are carotenoids. Carbon fixation pathways in prokaryotes and glycolysis and gluconeogenesis are believed to be involved in the synthesis of lutein and zeaxanthin (Figure S2). These genes, *ackA*, *pgm*, *gpmI*, and *pckA*, are unique in *Sphingomonas morindae* sp. NBD5 (Table 2). *gpmI* encodes a cofactor independent phosphoglycerate mutase that catalyzes the conversion of glycerate-3-phosphate to glycerate-2-phosphate. It involves in glycolysis/gluconeogenesis to generate glyceraldehyde 3-phosphate and pyruvate, providing a carbon skeleton for MP biosynthesis. *pckA* encodes a phosphoenolpyruvate carboxykinase, which catalyzes the conversion of oxaloacetate to phosphoenol-pyruvate. It participates to generate pyruvate in the glycolysis/gluconeogenesis pathway, providing a carbon skeleton for MP biosynthesis. *pgm* encodes a phosphoglucomutase directly involved in the glycolysis/gluconeogenesis pathway. It catalyzes α-D-glucose 1-phosphate into α-D-glucose 6-phosphate, which provides a carbon skeleton for MP biosynthesis. *ackA* encodes an acetate kinase that catalyzes the conversion of acetate to acetyl phosphate. It involves in the glycolysis/gluconeogenesis pathway, and the negative feedback regulates the process of pyruvate to acetyl CoA (Figure S3). The genes *pckG*, *porB*, *meh*, and *fldA* are characteristic in *Sphingopyxis* sp. USTB-05 (Table 2). *pckG* encodes phosphoenolpyruvate carboxykinase, which catalyzes the conversion of oxaloacetate to phosphoenol-pyruvate. It directly participates in the glycolysis/gluconeogenesis pathway to generate pyruvate and provide a carbon skeleton for MP biosynthesis. *porB* encodes 2-oxoglutarate ferredoxin oxidoreductase, which catalyzes the conversion of succinyl-CoA to 2-oxoglutarate in carbon fixation pathways. *meh* encodes 3-methylfumaryl-CoA hydratase, which catalyzes the conversion of 3-methylfumaryl-CoA to (3S)-citramalyl-CoA in the carbon fixation pathways in prokaryotes. *fldA* encodes 2-methylfumaryl-CoA isomerase, which catalyzes the conversion of 2-methylfumaryl-CoA to 3-methylfumaryl-CoA in the carbon fixation pathways. *pgm* exists in the NBD5 strain and encodes phosphoglucomutase, which catalyzes α-D-glucose 1-phosphate into α-D-glucose 6-phosphate. No gene encoding phosphoglucomutase was found in USTB-05 strain.

**Figure 4:**
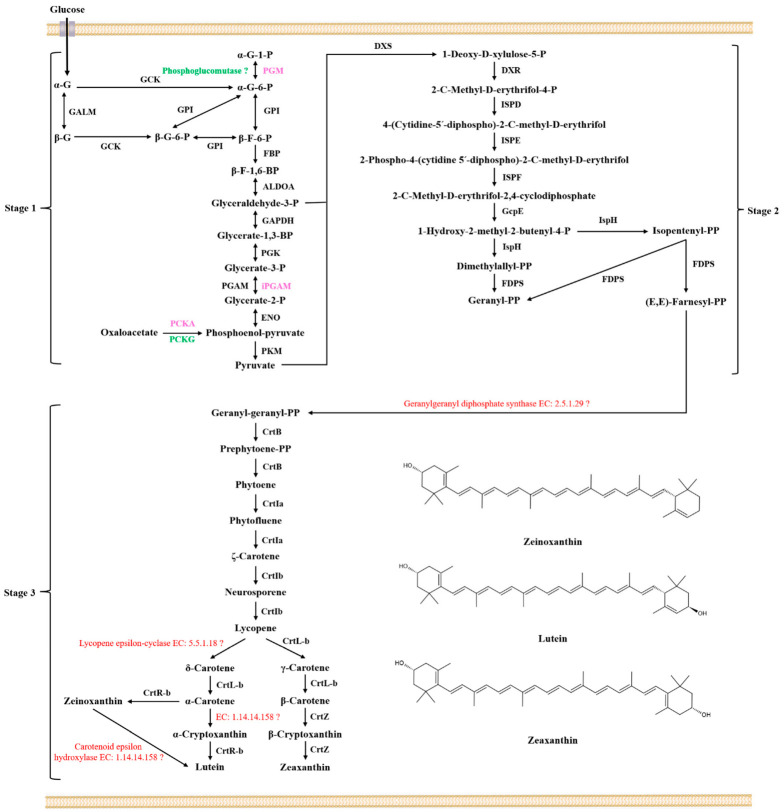
The proposed biosynthetic pathway of MP of *Sphingomonas morindae* sp. NBD5 and *Sphingopyxis* sp. USTB-05. α-G-1-P: α-D-glucose 1-phosphate; PGM: phosphoglucomutase; α-G-6-P: α-D-glucose 6-phosphate; GCK: glucokinase; α-G: α-D-glucose; GALM: aldose 1-epimerase; β-G: β-D-glucose; β-G-6-P: β-D-glucose 6-phosphate; GPI: glucose-6-phosphate isomerase; β-F-6-P: β-D-fructose 6-phosphate; FBP: fructose bisphosphatase; β-F-1,6-BP: β-D-fructose 1,6-bisphosphate; ALDOA: fructose bisphosphate aldolase; Glyceraldehyde-3-P: Glyceraldehyde-3-phosphate; GAPDH: glyceraldehyde-3-phosphate dehydrogenase; Glycerate-1,3-BP: Glycerate-1,3-bisphosphate; PGK: phosphoglycerate kinase; Glycerate-3-P: Glycerate-3-phosphate; PGAM: cofactor dependent phosphoglycerate mutase; iPGAM: cofactor independent phosphoglycerate mutase; Glycerate-2-P: Glycerate-2-phosphate; ENO: phosphopyruvate hydratase; PCKA: phosphoenolpyruvate carboxykinase; PCKG: phosphoenolpyruvate carboxykinase; PKM: pyruvate kinase; DXS: 1-deoxy-D-xylulose-5-phosphate synthase; DXR: 1-deoxy-D-xylulose-5-phosphate reductoisomerase; ISPD: 2-C-methyl-D-erythritol 4-phosphate cytidylyltransferase; ISPE: 4-(cytidine 5′-diphospho)-2-C-methyl-D-erythritol kinase; ISPF: 2-C-methyl-D-erythritol 2,4-cyclodiphosphate synthase; GcpE: (E)-4-hydroxy-3-methylbut-2-enyl-diphosphate synthase; IspH: 4-hydroxy-3-methylbut-2-en-1-yl diphosphate reductase; FDPS: dimethylallyltranstransferase; Dimethylallyl-PP: Dimethylallyl-bisphosphate; Isopentenyl-PP: Isopentenyl-bisphosphate; Geranyl-PP: Geranyl-bisphosphate; (E,E)-Farnesyl-PP: (E,E)-Farnesyl-bisphosphate; Geranyl-geranyl-PP: Geranyl-geranyl-bisphosphate; Prephytoene-PP: Prephytoene-bisphosphate; CrtB: 15-cis-phytoene synthase; CrtIa: phytoene desaturase; CrtIb: phytoene desaturase; CrtL-b: lycopene beta-cyclase; CrtR-b: beta-carotene hydroxylase; CrtZ: beta-carotene 3-hydroxylase. The black font in the figure indicates that enzymes or genes coexist in two strains, the red font in the figure indicates that enzymes or genes not found in two strains, the pink font indicates that enzymes or genes exist in *Sphingomonas morindae* sp. NBD5, and the green font indicates that enzymes or genes exist in *Sphingopyxis* sp. USTB-05.

The lutein and zeaxanthin synthesis genes in *Sphingomonas morindae* sp. NBD5 and *Sphingopyxis* sp. USTB-05 genomes mainly exist in the terpenoid backbone biosynthesis pathway and the carotenoid biosynthesis pathway, which are roughly the same (Figures S4 and S5). Both strains are the presence of β-carotene 3-hydroxylase, which is involved in the last step of lutein and zeaxanthin synthesis, and its corresponding code gene is *crtR*. This is a fact that lycopene exists as a synthetic intermediate, which is synthesized through glycolysis pathway, terpenoid backbone biosynthesis pathway and carotenoid biosynthesis pathway (Figure 4).

### 3.5. Identification of Lutein of Sphingomonas morindae sp. NBD5 and Sphingopyxis sp. USTB-05

Under the applied chromatographic conditions, the retention time of standard lutein was at 5.5 min on LC-Q-TOF/MS (Figure S6a). A peak in the extract of *Sphingomonas morindae* sp. NBD5 also arose at the same retention time (Figure S6b). Furthermore, the scanning profiles from 350 nm to 550 nm between lutein peak and a peak in the extract of *Sphingomonas morindae* sp. NBD5 at same retention time were almost same each other and the maximum absorbance were all at 446 nm or so (Figure S6). The mass spectral analysis of standard lutein (Figure S7a) and a peak at same retention time in the extract of *Sphingomonas morindae* sp. NBD5 (Figure S7b) revealed a major ion at *m/z* 568.4289, corresponding to the [M+H]^+^ protonated molecular ion, which all accorded with the lutein molecular weight [21,22,23]. The *m/z* 568.4289 was selected as the parent ion, and the secondary scan was performed. In the detection of standard lutein and the extract of *Sphingomonas morindae* sp. NBD5, the parent ion *m/z* 568.4283 was subjected to a collision cracking of 15 eV in the collision cell, where in the main ions were *m/z* 476.3656 [M+H-92]^+^, *m/z* 458.3542 [M+H-92-18]^+^ and *m/z* 430.3239 [M+H-92-18-28]^+^ (Figure S8). Among them, *m/z* 476.3656 is formed when the molecular ion peak loses the carotenoid characteristic group of *m/z* 92 [22]. Corresponding with the data in the report, the molecular formula of *m/z* 476.3656 characteristic ion fragment is C_33_H_48_O_2_, which is formed by the loss of C_7_H_8_ molecule by the parent ion *m/z* 568.4280 [21], which completely confirmed that lutein could be produced by *Sphingomonas morindae* sp. NBD5.

**Figure 5:**
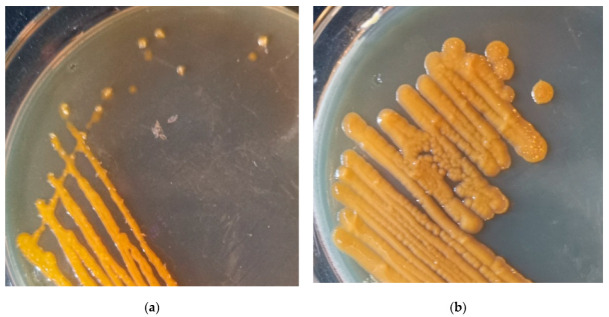
(**a**) The streak plate colonies of *Sphingomonas morindae* sp. NBD5 grown on solid medium at five day; (**b**) the streak plate colonies of *Sphingopyxis* sp. USTB-05 grown on solid medium at five day.

The streak plate colonies of *Sphingomonas morindae* sp. NBD5 grew on solid medium at five days. The yellow color of *Sphingomonas morindae* sp. NBD5 colonies is similar to that of lutein (Figure 5a). In conclusion, genomic annotation, mass spectrometry identification, and appearance comparison showed that strains of *Sphingomonas morindae* sp. NBD5 produced lutein. Similarly, the genomic annotation and appearance comparison of *Sphingopyxis* sp. USTB-05 showed that *Sphingopyxis* sp. USTB-05 produced lutein or zeaxanthin (Figure 4 and Figure 5b).

## 4. Discussion

*Sphingomonas morindae* sp. NBD5 had two genomes, which were relatively rare in *Sphingomonas*. Two genomes and two plasmids may have special significance in the biosynthesis and regulation of macular pigment of this strain. Both plasmids were stable in the strain, indicating that they have different replicons. Two genomes have been reported in eukaryotes, and this heterokaryosis plays a role in the adaptation of arbuscular mycorrhizal fungi to different plant hosts [24]. *Sphingomonas morindae* sp. NBD5 strain is an endophyte from Noni fruit, and the two genomes may play a key role in its adaptation in the host. Recent studies prove that genome-wide phylogeny can improve the phylogenetic accuracy and preferably delimit the species borderlines [25]. As of 7 May 2022, 127 strains of *Sphingomonas* have published genome data in the National Center for Biotechnology Information (NCBI) database. *Sphingomonas morindae* sp. NBD5 whole-genome sequencing enriches the whole genome data of MP-producing bacterium.

Lutein and zeaxanthin biosynthesis can be divided into three stages: basic components formation, components condensation, and skeleton structure assembly (Figure 4). Synthetase genes related to lutein and zeaxanthin are discovered in the glycolysis/gluconeogenesis pathway, terpenoid skeleton biosynthesis pathway and carotenoid biosynthesis pathway (Figures S3–S5). In the first stage, glyceraldehyde 3-phosphate (G3P) and pyruvate are synthesized via the glycolysis pathway (Figure S3). In the glycolysis pathway, the glucose was transferred into the bacteria through the receptors on the surface of the bacteria in two strains. All enzymes involved in the conversion of α-D-glucose 1-phosphate to pyruvate were found in two strains. PGM, iPGAM, PCKA are three unique enzymes in the genome of *Sphingomonas morindae* sp. NBD5. PCKG is a unique enzyme in the genome of *Sphingopyxis* sp. USTB-05. PGM is α-D-glucose-1,6-bisphosphate dependent phosphoglucomutases. iPGAM is 2,3-diphosphoglycerate independent phosphoglycerate mutase. In particularly, iPGAM shows the greatest activity in the absence of 2,3-diphosphoglycerate in Gram-positive bacteria and is rarely reported in Gram-negative bacteria [26].

In the second stage, the 2-C-methyl-D-erythritol 4-phosphate (MEP) pathway, also known as the 1-deoxy-D-xylulose 5-phosphate (DXP) or non-mevalonate pathway [27]. DXS, DXR, ISPD, ISPE, ISPF, GcpE, ISPH, and FDPS are annotated in the genome of *Sphingomonas morindae* sp. NBD5 and *Sphingopyxis* sp. USTB-05 (Figure S4). DXS catalyzes the condensation of pyruvate and glyceraldehyde 3-phosphate to form 1-deoxy-D-xylulose 5-phosphate. DXS is an open reading frame-encoding enzyme on the chromosomal map of *E. coli*. Cloning of *dxs* was overexpressed and purified to produce a specific activity of enzyme protein of 0.85 units per mg, and DXS may be widespread in bacteria and plant chloroplasts [28]. The *dxs* gene was cloned from *Streptomyces* sp. CL190 and *E. coli*, respectively. The overexpressed and purified experiments show that they have the same enzymatic properties although they have different origins [29]. DXR catalyses transform 1-deoxy-D-xylulose 5-phosphate into 2-C-methyl-D-erythritol 4-phosphate. DXR is an NADPH-dependent enzyme that also requires metal ions (Mn^2+^, Co^2+^ or Mg^2+^) with Mn^2+^ being the most efficient. The reaction mechanism of this enzyme is shown to be a retro-aldol/aldol reaction [30]. Earlier experiments, overexpressing the recombinant gene *yaeM* corresponding to DXR in *E. coli* is found that the enzyme can convert 1-deoxy-D-xylulose 5-phosphate to 2-C-methyl-D-erythritol 4-phosphate through one-step intramolecular rearrangement and reduction. This result suggests that DXR is responsible for terpenoid biosynthesis in *E. coli* [31]. ISPD catalyzes the conversion of 2-C-methyl-D-erythrifol 4-phosphate to 4-(cytidine-5′-diphosphate)-2-C-methyl-D-erythrifol. A new enzyme, ISPD, was discovered in *E. coli* [32]. These genes *ygbP* and *ygbB* corresponding to ISPD may be involved in this step of transformation [33]. ISPE catalyses transform 4-(cytidine-5′-diphosphate)-2-C-methyl-D-erythrifol into 2-phospho-4-(cytidine 5′-diphospho)-2-C-methyl-D-erythrifol. A new enzyme, 4-(cytidine 5′-diphospho)-2-C-methyl-D-erythritol kinase (ISPE), was discovered in *E. coli* [34]. Earlier experiments, overexpressing the gene *ychB* corresponding to ISPE in *E. coli* and combining with isotopic labeling, found the catalytic activity of ISPE. The predicted protein sequence similarity between YchB and tomato cDNA pTOM41 was 30%, and this phenomenon was related to the transformation of chloroplast to chromosome [35]. ISPF catalyzes the conversion of 2-phospho-4-(cytidine 5′-diphospho)-2-C-methyl-D-erythrifol to 2-C-methyl-D-erythritol 2,4-cyclodiphosphate. A new enzyme, ISPF, was discovered in *E. coli* [36]. Earlier experiments, overexpressing the gene *ygbB* corresponding to ISPF in *E. coli* and combining with isotopic labeling, found that the catalytic activity of ISPF [37]. GcpE catalyses transform 2-C-methyl-D-erythritol 2,4-cyclodiphosphate into 1-hydroxy-2-methyl-2-butenyl-4-diphosphate. Combining database information and heterologous expression of *gcpE* and *petF* genes indicated that PetF could transfer electrons to GcpE [38]. Experimental results indicate that GcpE is a ferredoxin-dependent enzyme with [4Fe-4S] cluster [39]. In bacteria, GcpE relies on NADPH/flavodoxin/flavodoxin reductase as a reductive shuttle system for electron transfer [40]. ISPH catalyzes the conversion of 1-hydroxy-2-methyl-2-butenyl-4-diphosphate to isopentenyl diphosphate (IPP) and dimethylallyl diphosphate (DMPP). Expression of the ispH (*lytB*) gene in *E. coli* converts 1-hydroxy-2-methyl-2-butenyl-4-diphosphate to IPP and DMPP [41]. Further studies reveal that the enzyme possesses molecular oxygen-sensitive [4Fe-4S] clusters [42]. Overexpression of the *ispH* gene in *E. coli* using the isc operon resulted in at least a 200-fold increase in the catalytic activity of the purified protein ISPH [43]. FDPS catalyses transform DMPP or IPP into geranyl diphosphate (GPP), and also catalyses transform IPP into farnesyl diphosphate (FPP). Previous studies have obtained isoprenyltransferase from *Micrococcus lysodeikticus*, which catalyzes the synthesis of all-trans GPP from FPP and IPP [44]. FPP synthase, obtained from *Bacillus subtilis*, catalyzes the conversion of IPP and DMPP or GPP to all-trans FPP. The metal ions Mg^2+^ or Mn^2+^ are crucial to the catalytic activity, but Mn^2+^ is less effective [45].

Geranylgeranyl diphosphate synthase is the most important to the initiation of the carotenoid synthesis pathway, producing the only product geranylgeranyl diphosphate (GGPP). This study has not annotated synthase (EC: 2.5.1.29) in the existing database. We predict that there are new synthases with lower homology in the genome of *Sphingomonas morindae* sp. NBD5 and *Sphingopyxis* sp. USTB-05. One molecule of FPP condenses with one molecule IPP to form GGPP under the catalysis of CrtE in *Erwinia uredovora* and *Agrobacterium aurantiacum* [46]. The GGPP synthase genes were screened from the genomic DNA libraries of archaebacterial *Sulfolobus acidocaldarius* and *Streptomyces acidocaldarius*. Heterologous expression showed that DMPP or GPP were used as substrates producing GGPP [46]. GGPP synthase (EC: 2.5.1.29) was purified from *Methanobacterium thermoformicicum* SF-4. It is a dimeric protein consisting of two identical subunits and is stable upon treatment at 65 °C for 30 min [47]. GGPP synthase from *Erwinia uredovora* is overexpressed in *E. coli*, reaction rates and Km values indicate that GPP and FPP are allyl substrates for GGPP synthase, but not DMPP [48]. Comparing the substrate specificity of GGPP synthases from *Micrococcus lysodeikticus* and pumpkin seedling, it is found that the enzyme in pumpkin seedling has the highest activity, while the lowest activity is in *Micrococcus lysodeikticus* [49]. It is consistent with the analysis results that GGPP synthases are derived from different organisms with widely different properties.

In the third stage, GGPP is transferred to lutein and zeaxanthin in carotenoid biosynthesis pathways (Figure 4). Six out of at least nine enzymes involved in the lutein and zeaxanthin biosynthesis are annotated into the genomes of two strains (Figure S5). CrtB catalyzes the conversion of GGPP to prephytoene diphosphate. Evidence for the presence of the *crtB* and the *crtB* operon was found in the purple photosynthetic bacterium *Rhodobacter sphaeroides* [50]. At the same time, ORF-A protein homologous to the amino acid sequence of CrtB was found in the *Thernus thermophilus*, heterologous expression showed that the strain containing ORF-A protein had three times the carotenoid production of the strain without containing ORF-A protein [51]. Further experiments showed that the CrtB enzyme in *Erwinia uredovora* requires Mn^2+^ and had an optimum pH of 8.2 to produce the only product phytoene. CrtB is a highly conserved enzyme that can be used to design new drugs or pesticides with specific targets [52]. 

Two desaturases are annotated in the genomic data of *Sphingomonas morindae* sp. NBD5 and *Sphingopyxis* sp. USTB-05. CrtIa catalyzes the conversion of phytoene in the cis conformation to ζ-carotene in two steps. CrtIb catalyzes the conversion of ζ-carotene in the trans conformation to lycopene. The complete *crtI* gene of *Erwinia* was heterologously expressed in *E. coli*, and the purified enzyme could catalyze the conversion of cis-phytoene into trans-lycopene as well as to bisdehydrolycopene [53]. The complete *crtI* gene was overexpressed in *E. coli*, *Rhodobacter capsulatus*, and *Rhodobacter sphaeroides*, which catalyzed the desaturation of phytoene to produce neurosporene. It is an ATP-dependent enzyme [50,54]. Further studies found that there were two different forms of CrtIa and CrtIb in *Myxococcus xanthus*. CrtIa catalyzes the dehydrogenation of carotene in the cis conformation, and CrtIb catalyzes the dehydrogenation of carotene in the trans conformation [55].

Two cyclases are found in the genomic data of *Sphingomonas morindae* sp. NBD5 and *Sphingopyxis* sp. USTB-05. CrtL-b is responsible for three step cyclization reaction. Unannotated enzymes catalyze the conversion of lycopene to δ-carotene (Figure 4). CrtL-b catalyzes the cyclization of lycopene to form different types of carotenes. Earlier reports showed that CrtL catalyzed the conversion of acyclic lycopene to bicyclic β-carotene [56]. The predicted lycopene cyclases in rice are CrtL-b and CrtL-e proteins. They are only about 34% and 50% similar to CrtL from *Synechococcus* sp. PCC 7942 [56]. Other types of lycopene cyclases are reported, lycopene cyclase genes from *Erwinia* (*crtY*) or the plant capsicum (*lcy*) can catalyze monocyclic β-carotene produce bicyclic carotenoids 7,8-dihydro-β-carotene [57]. A fourth class of lycopene cyclases are found in photosynthetic bacteria, CruA and CruP exhibit lycopene cyclase activity [58]. Two separate Lycopene epsilon-cyclases (EC: 5.5.1.18) are identified for the first time in the marine cyanobacterium *Prochlorococcus marinus* MED4. The enzyme crtL-e is responsible for the formation of cyclic carotenoids with β- or ε-terminal groups [59]. Although the cyclase crtL-e is not annotated in *Sphingomonas morindae* sp. NBD5 and *Sphingopyxis* sp. USTB-05, new genes distantly related to this cyclase is found based on the overall metabolic process.

Three hydroxylases are found in the genomic data of *Sphingomonas morindae* sp. NBD5 and *Sphingopyxis* sp. USTB-05. CrtR-b and CrtZ are responsible for four steps hydroxylation. Unannotated enzymes catalyze the conversion of zeinoxanthin to lutein and α-carotene to α-cryptoxanthin (Figure 4). CrtR-b catalyzes the conversion of α-Carotene to zeinoxanthin and α-Cryptoxanthin to lutein, respectively. Recently, this enzyme is found in the chloroplasts of tomato, tobacco and *Haematococcus pluvialis* [60]. It is an oxidoreductase, with reduced iron-sulfur protein as one donor, which can introduce hydroxyl groups to the donor molecule [60]. CrtZ catalyzes the conversion of β-carotene to zeaxanthin, β-carotene hydroxylase (CrtZ) acts as a hydroxylase. Meanwhile, β-carotene hydroxylase (CrtZ) is reported to be the rate-limiting enzyme in astaxanthin biosynthesis [61]. In eubacteria and cyanobacteria, multiple genes encoding β-carotene 3-hydroxylase, which are responsible for the conversion of β-carotene to zeaxanthin [61]. Furthermore, CrtZ adds a hydroxyl group to the 7,8-dihydro-β end group and the β end group in *Erwinia* [57]. There are also other types of tomato erythromycin cyclases have been reported to be CHXB and CHXE during the carotenoid biosynthesis in strawberry. The enzyme CHXB is responsible for the conversion of α-carotene to zeinoxanthin, β-carotene to β-cryptoxanthin and β-cryptoxanthin to zeaxanthin. The enzyme CHXE is responsible for catalyzing the conversion of zeinoxanthin to lutein [62]. The homology and other characteristics of CrtZ, CHXB and CHXE need be further clarified. The carotenoid epsilon-hydroxylase (EC: 1.14.14.158) is found in the cytochrome enzyme family in *Arabidopsis thaliana*, and it is named the LUT1 responsible for catalyzing the conversion of zeinoxanthin to lutein. Although the hydroxylase LUT1 is not annotated in these two strains in this study, a new carotenoid epsilon-hydroxylase or gene may be discovered based on the prediction of metabolic process [63].

The MEP pathway is normally present in most bacteria, green algae and plant plastids. However, IPP and FPP molecules are also produced via the mevalonate (MVA) pathway in archaea, fungi, higher plant cytoplasm and other eukaryotes [64,65]. In watercress and microalgae, the key steps are the conversion of three IPP molecules and DMPP by the enzyme (GGPPS/GGPS/FPPS) to generate GGPP by the condensation process [9,11,26]. In living organisms, the condensation of IPP with DMPP to form GPP is catalyzed by geranyl diphosphate synthase (GPS). Finally, GPP is condensed with one molecule of IPP to form FPP by farnesyl diphosphate synthase (FPS) [66]. In eukaryotes, especially higher plants, lutein and zeaxanthin are primarily synthesized from terpenoids. Two molecules of GGPP are used to synthesize phytoene. Phytoene is then converted to ζ-carotene, after which ζ-carotene is converted into lycopene. Lycopene is then converted to α-carotene by lycopene cyclase (εLCY/βLCY). Finally, the formation of lutein from α-carotene is catalyzed by α-carotene hydroxylase (βCHX/εCHX) [67].

Recently, the biosynthetic pathway from GGPP to zeaxanthin is also found in various organisms, such as: *Echinicola marina*, *Acaryochloris*, *Prochlorococcus* and *Synechococcus* PCC7942 [12,65,68,69]. *Sphingomonas* sp. SG73 is the same genus as *Sphingomonas morindae* sp. NBD5, the biosynthetic pathway from zeaxanthin to nostoxanthin is further discovered [70]. Based on Liquid Chromatography-UltraViolet-Mass Spectrometry (LC–UV-MS/MS) analysis, three biosynthetic pathways from GGPP to lutein, zeaxanthin, diketospirilloxanthin are discovered in *Flavobacterium* [13]. Further, we optimized the strain NBD5 fermentation culture by temperature, carbon source and nitrogen source, and obtained lutein yield of 1.6 mg/g, 1.8 mg/g and 1.9 mg/g, respectively [15]. In this article, the biosynthetic pathways of lutein and zeaxanthin are obtained from the genome annotation data of *Sphingomonas morindae* sp. NBD5 and *Sphingopyxis* sp. USTB-05. They are consistent with the above metabolic mass spectrometry data analysis results. We further clarified the MP biological enzymes involved in the reaction and the corresponding coding genes.

## 5. Conclusions

The whole genome of *Sphingomonas morindae* sp. NBD5 consists of two circular chromosomes of 4,239,716 bp with 3882 protein coding genes including 59 protein-coding genes related to MP biosynthesis, of which four genes (*ackA*, *pgm*, *gpmI* and *pckA*) are unique. These genes, *pckG*, *porB*, *meh*, and *fldA*, are unique in *Sphingopyxis* sp. USTB-05. Lutein and zeaxanthin synthesis metabolic pathways and synthetic genes are discovered in *Sphingomonas morindae* sp. NBD5 and *Sphingopyxis* sp. USTB-05. Three new enzymes are found in whole metabolic pathway, they are GGPP synthase, lycopene epsilon-cyclase, carotenoid epsilon hydroxylase. This study expands the understanding of the pathway for complete biosynthesis of MP by *Sphingomonas morindae* sp. NBD5 and *Sphingopyxis* sp. USTB-05.

## Figures and Tables

**Table 1:** Genome features of *Sphingomonas morindae* sp. NBD5.

Category	Value
Genome size (bp)	4,239,716
tmRNA	1
tRNA	61
Protein-coding sequences (CDSs)	3882
chromosome G + C content (%)	70%
plasmid	2
plasmid G + C content (%)	63%

**Table 2:** Genes related to macular pigment biosynthesis in *Spingomonas morindae* sp. NBD5 and *Sphingopyxis* sp. USTB-05.

Pathway	Genes	Coded Protein/Enzyme	Catalytic Substrate	Catalytic Product
Glycolysis&Gluconeogenesis	***pgm*** *	Phosphoglucomutase	α-D-glucose 1-phosphate	α-D-glucose 6-phosphate
	*glk*	Glucokinase	α-D-glucose	α-D-glucose 6-phosphate
	*galM*	Aldose 1-epimerase	α-D-glucose	β-D-glucose
	*pgi*	Glucose-6-phosphate isomerase	α-D-glucose 6-phosphate; β-D-glucose 6-phosphate	β-D-fructose 6-phosphate
	*fbp*	Fructose bisphosphatase	β-D-fructose 6-phosphate	β-D-fructose 1,6-bisphosphate
	*fbaB*	Phosphoglycerate kinase	β-D-fructose 1,6-bisphosphate	Glyceraldehyde-3-phosphate; glycerone-phosphate
	*tpiA*	Phosphotriose isomerase	Glycerone-phosphate	Glyceraldehyde-3-phosphate
	*gapA*	Glyceraldehyde-3-phosphate dehydrogenase	Glyceraldehyde-3-phosphate	Glycerate-1,3-bisphosphate
	*pgk*	Phosphoglycerate kinase	Glycerate-1,3-bisphosphate	Glycerate-3-phosphate
	*gpmA*	Cofactor dependent phosphoglycerate mutase	Glycerate-3-phosphate	Glycerate-2-phosphate
	***gpmI*** *	Cofactor independent phosphoglycerate mutase	Glycerate-3-phosphate	Glycerate-2-phosphate
	***pckA*** *	Phosphoenolpyruvate carboxykinase	Oxaloacetate	Phosphoenol-pyruvate
	***pckG*** ^#^	Phosphoenolpyruvate carboxykinase	Oxaloacetate	Phosphoenol-pyruvate
	*eno*	Phosphopyruvate hydratase	Glycerate-2-phosphate	Phosphoenol-pyruvate
	*pykF*	Pyruvate kinase	Phosphoenol-pyruvate	Pyruvate
	*korA*	2-oxoacid oxidoreductase	Pyruvate	Acetyl-CoA
	*aceF*	Dihydrolipoate acetyltransferase	S-Acetyl-dihydrolipoamide-E	Acetyl-CoA
	*aceE*	Pyruvate dehydrogenase	Pyruvate	S-Acetyl-dihydrolipoamide-E
	*lpd*	Dihydrolipoyl dehydrogenase	Dihydrolipoamide-E	Lipoamide-E
	*sapD*	Propionaldehyde dehydrogenase	Acetate	Acetaldehyde
	*frmA*	Ethanol dehydrogenase	Acetaldehyde	Ethanol
Carbon fixation pathways in prokaryotes	*acs*	Acetate-CoA ligase	Acetate	Acetyl-CoA
	***ackA*** *	Acetate kinase	Acetate	Acetyl phosphate
	*accA*	Acetyl-CoA carboxylase	Acetyl-CoA	Malonyl-CoA
	*yqeF*	Acetyl-CoA acetyltransferase	Acetoacetyl-CoA	Acetyl-CoA
	*acnA*	Cis-aconitase	Cis-Aconitate	Citrate
	*icd*	Oxalosuccinate decarboxylase	2-oxoglutarate	Isocitrate
	***porB*** ^#^	2-oxoglutarate ferredoxin oxidoreductase	Succinyl-CoA	2-oxoglutarate
	*ppdK*	Pyruvate-phosphate ligase	Pyruvate	Phosphoenol-pyruvate
	*ppc*	Phosphoenolpyruvate carboxylase	Phosphoenol-pyruvate	Oxaloacetate
	*mdh*	Malate dehydrogenase	Oxaloacetate	(S)-malate
	*fumA*	Fumarate hydratase	(S)-malate	Fumarate
	*sdhA*	Succinate dehydrogenase	Fumarate	Succinate
	*sdhB*	Succinate dehydrogenase	Fumarate	Succinate
	*sucC*	Succinate-CoA ligase	Succinate	Succinyl-CoA
	*metF*	Methylenetetrahydrofolate reductase	5,10-Methylene-THF	5-Methyl-tetrahydrofolate
	*folD*	Methylenetetrahydrofolate dehydrogenase	5,10-Methenyl-THF	5,10-Methylene-THF
	*mce*	Methylmalonyl-CoA epimerase	(S)-Methylmalonyl-CoA	(R)-Methylmalonyl-CoA
	*scpA*	Methylmalonyl-CoA mutase	(R)-Methylmalonyl-CoA	Succinyl-CoA
	***meh*** ^#^	3-methylfumaryl-CoA hydratase	3-Methylfumaryl-CoA	(3S)-Citramalyl-CoA
	***fldA*** ^#^	2-methylfumaryl-CoA isomerase	2-Methylfumaryl-CoA	3-Methylfumaryl-CoA
Terpenoid backbone biosynthesis	*atoB*	Acetyl-CoA C-acetyltransferase	Acetyl-CoA	Acetoacetyl-CoA
	*dxs*	1-deoxy-D-xylulose-5-phosphate synthase	Glyceraldehyde-3-phosphate; pyruvate	1-deoxy-D-xylulose-5-phosphate
	*dxr*	1-deoxy-D-xylulose-5-phosphate reductoisomerase	1-deoxy-D-xylulose-5-phosphate	2-C-methyl-D-erythritol 4-phosphate
	*ispD*	2-C-methyl-D-erythritol 4-phosphate cytidylyltransferase	2-C-methyl-D-erythritol 4-phosphate	4-(cytidine 5′-diphospho)-2-C-methyl-D-erythritol
	*ispE*	4-(cytidine 5′-diphospho)-2-C-methyl-D-erythritol kinase	4-(cytidine 5′-diphospho)-2-C-methyl-D-erythritol	2-Phospho-4-(cytidine 5′-diphospho)-2-C-methyl-D-erythritol
	*ispDF*	2-C-methyl-D-erythritol 2,4-cyclodiphosphate synthase	2-Phospho-4-(cytidine 5′-diphospho)-2-C-methyl-D-erythritol	2-C-methyl-D-erythritol 2,4-cyclodiphosphate
	*gcpE*	(E)-4-hydroxy-3-methylbut-2-enyl-diphosphate synthase	2-C-methyl-D-erythritol 2,4-cyclodiphosphate	1-Hydroxy-2-methyl-2-butenyl 4-diphosphate
	*ispG*	(E)-4-hydroxy-3-methylbut-2-enyl-diphosphate synthase	2-C-methyl-D-erythritol 2,4-cyclodiphosphate	1-Hydroxy-2-methyl-2-butenyl 4-diphosphate
	*ispH*	4-hydroxy-3-methylbut-2-en-1-yl diphosphate reductase	1-Hydroxy-2-methyl-2-butenyl 4-diphosphate	Isopentenyl-PP or Dimethylallyl-PP
	*ispA*	Geranyl pyrophosphate synthase	Isopentenyl-PP or Dimethylallyl-PP	Geranyl-PP
	*ispB*	Octaprenyl-diphosphate synthase	(E,E)-Famesyl-PP	Octaprenyl-diphosphate
	*ispU*	Undecaprenyl diphosphate synthetase	(E,E)-Famesyl-PP	Ditrans, polycis-undecaprenyl diphosphate
Carotenoid biosynthesis	*crtB*	15-cis-phytoene synthase	Geranyl-geranyl-PP	Prephytoene-PP
	*crtE*	Phytoene desaturase	15-cis-phytoene	All-trans-neurosporene
	*crtI*	Phytoene desaturase	Phytoene	Lycopene
	*crtQ*	Phytoene desaturase	15-cis-phytoene	All-trans-lycopene
	*crtL*	Lycopene β-cyclase	Lycopene	γ-Carotene
	*crtR*	β-carotene 3-hydroxylase	β-Carotene	Zeaxanthin

* The bold fonts indicate that these genes are only present in *Spingomonas morindae* sp. NBD5 genome, but not in *Sphingopyxis* sp. USTB-05. ^#^ The bold fonts indicate that these genes are only present in *Sphingopyxis* sp. USTB-05 genome, but not in *Spingomonas morindae* sp. NBD5.

## Data Availability

The data presented in this study are available in article https://doi.org/10.3390/toxins14050333.

## Supplementary Materials

The following supporting information can be downloaded at: https://www.mdpi.com/article/10.3390/microorganisms11020266/s1, Figure S1: Sequence distribution of data lengths of nanopore sequencing; Figure S2: Comparison of genes related to lutein synthesis in the genomes of *Sphingomonas morindae* sp. NBD5 and *Sphingopyxis* sp. USTB-05; Figure S3: Comparison of genes related to glycolysis and gluconeogenesis pathway in the genomes of *Sphingomonas morindae* sp. NBD5 and *Sphingopyxis* sp. USTB-05; Figure S4: Comparison of genes related to terpenoid backbone biosynthesis in the genomes of *Sphingomonas morindae* sp. NBD5 and *Sphingopyxis* sp. USTB-05; Figure S5: Comparison of genes related to carotenoid biosynthesis in the genomes of *Sphingomonas morindae* sp. NBD5 and *Sphingopyxis* sp. USTB-05; Figure S6: (a) Peaks and scanning profiles of standard lutein; (b) Peaks and scanning profiles of the extract of *S. morindae* NBD5 on LC-Q-TOF/MS [15]; Figure S7: (a) First-order mass spectrometry of standard lutein on LC-Q-TOF/MS; (b) First-order mass spectrometry of the extract of *S. morindae* NBD5 on LC-Q-TOF/MS [15]; Figure S8: (a) Second-order mass spectrometry of standard lutein on LC-Q-TOF/MS; (b) Second-order mass spectrometry of the extract of *S. morindae* NBD5 on LC-Q-TOF/MS [15]; Table S1: Gene Ontology (GO) classifications of genes; Table S2: Clusters of Orthologous Group (COG) classifications of genes.
