# Supplementary material for: Comparative Genomic Analysis of Sphingomonas morindae sp. NBD5 and Sphingopyxis sp. USTB-05 for Producing Macular Pigment
Source: Microorganisms. 2023 Jan 19;11(2):266. doi: 10.3390/microorganisms11020266 (PMC9967899; doi:10.3390/microorganisms11020266)
Supplement: Supplementary file 1 [file microorganisms-11-00266-s001.zip › Figures S1-S5.pptx]

## Slide 1
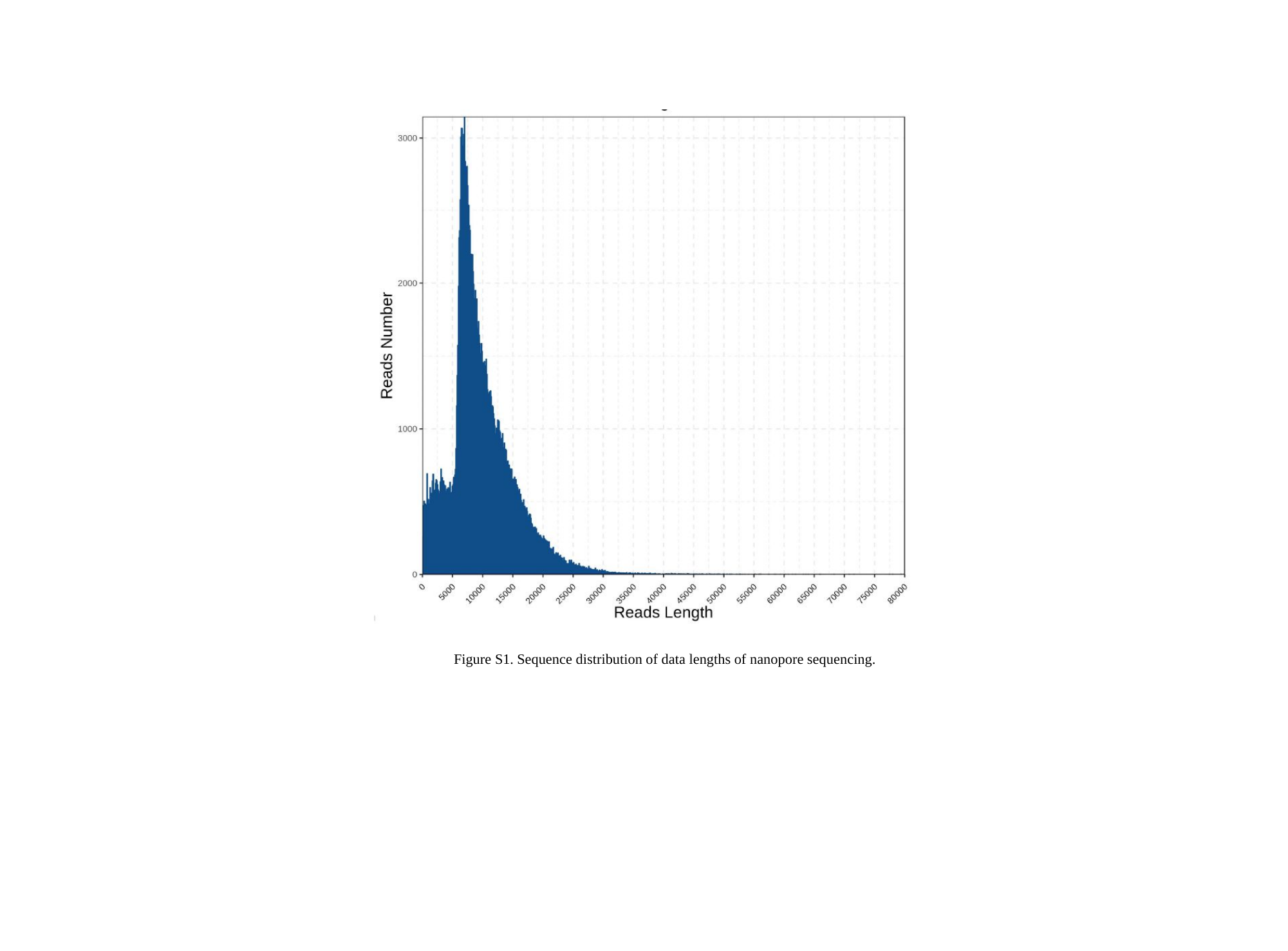

Figure S1. Sequence distribution of data lengths of nanopore sequencing.

## Slide 2
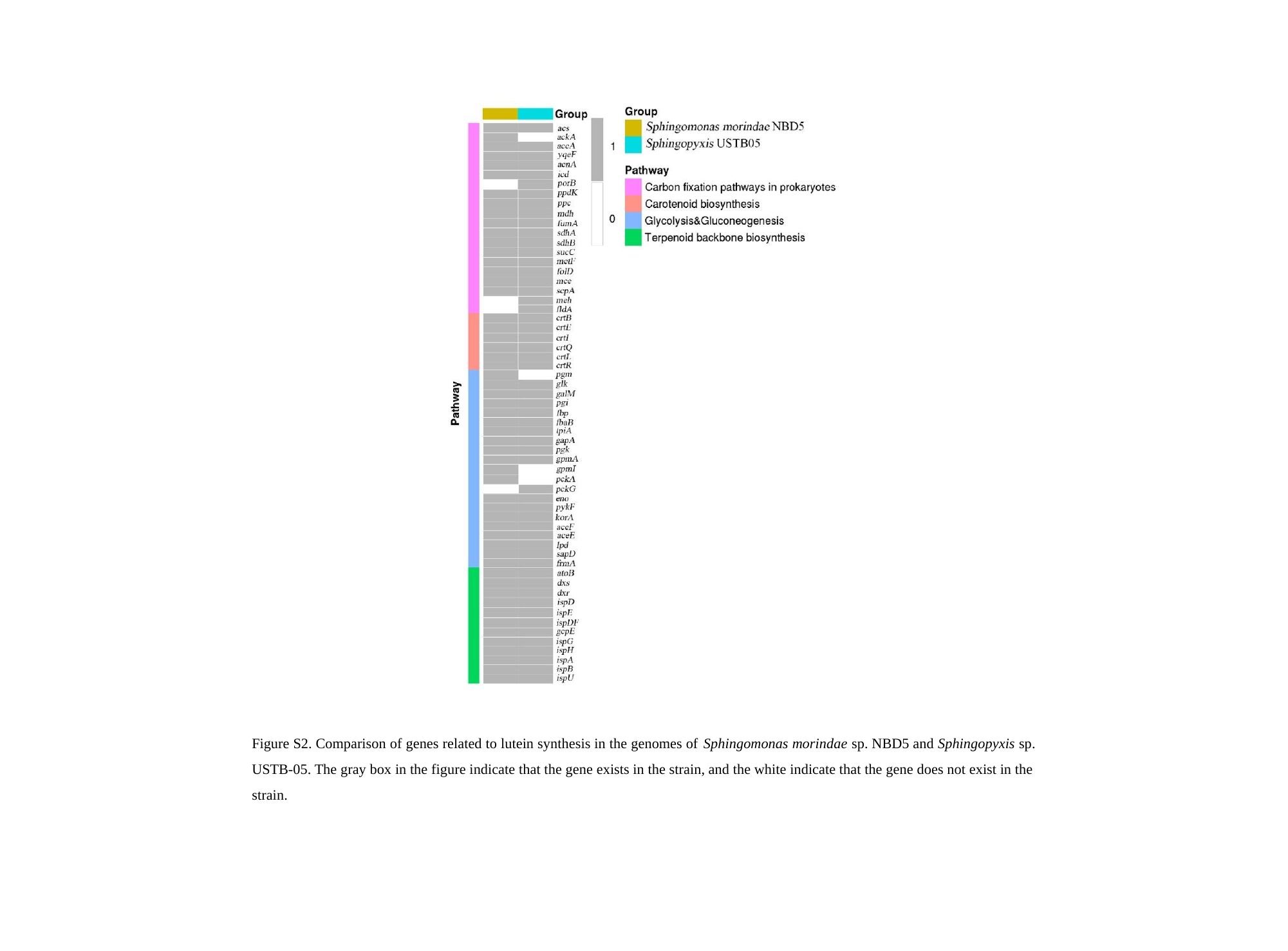

Figure S2. Comparison of genes related to lutein synthesis in the genomes of Sphingomonas morindae sp. NBD5 and Sphingopyxis sp. USTB-05. The gray box in the figure indicate that the gene exists in the strain, and the white indicate that the gene does not exist in the strain.

## Slide 3
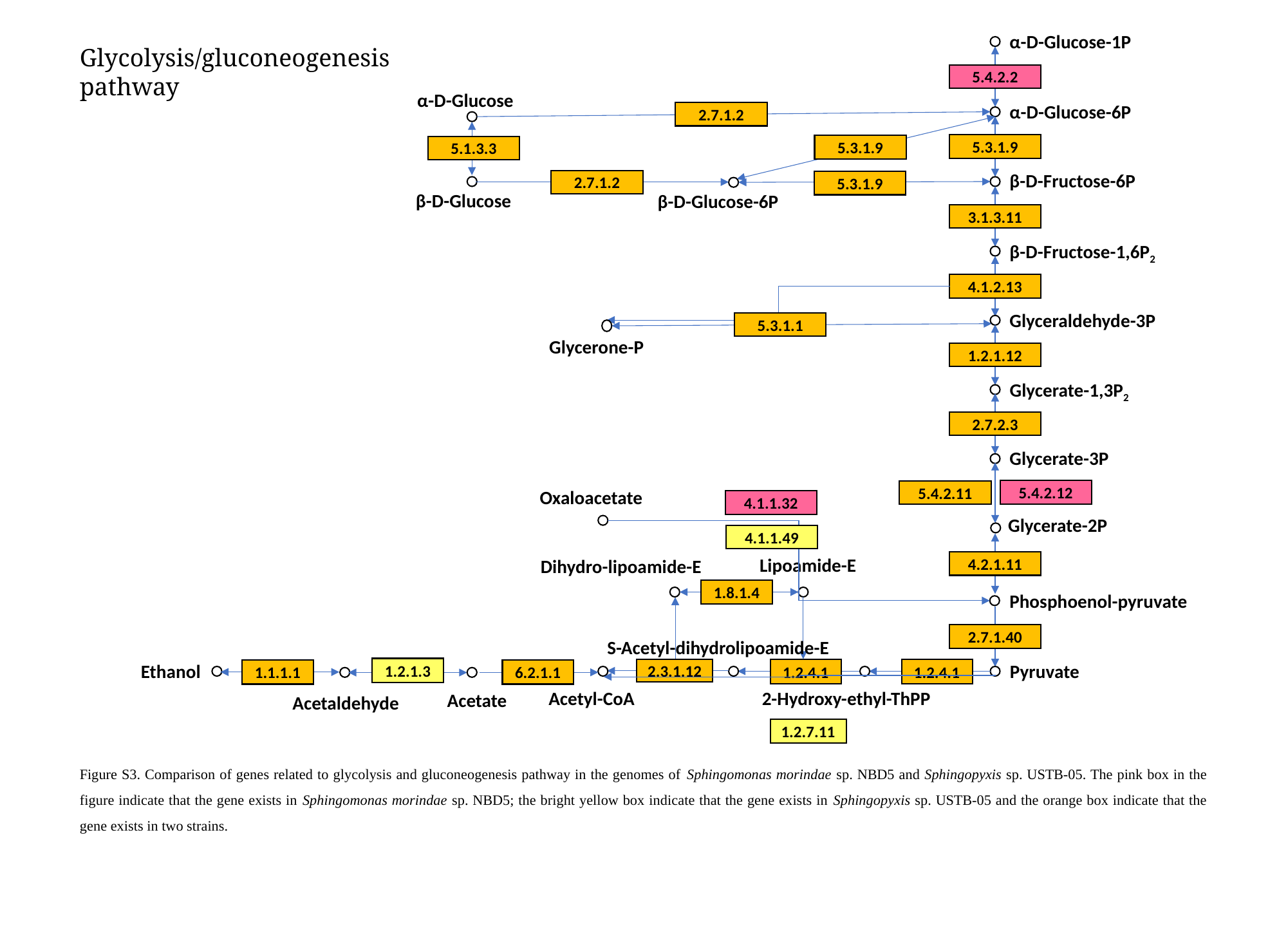

α-D-Glucose-1P
5.4.2.2
α-D-Glucose
α-D-Glucose-6P
2.7.1.2
5.3.1.9
5.3.1.9
5.1.3.3
β-D-Fructose-6P
2.7.1.2
5.3.1.9
β-D-Glucose
β-D-Glucose-6P
3.1.3.11
β-D-Fructose-1,6P2
4.1.2.13
Glyceraldehyde-3P
5.3.1.1
1.2.1.12
Glycerone-P
Glycerate-1,3P2
2.7.2.3
Glycerate-3P
5.4.2.12
5.4.2.11
Oxaloacetate
4.1.1.32
Glycerate-2P
4.1.1.49
4.2.1.11
Lipoamide-E
Dihydro-lipoamide-E
1.8.1.4
Phosphoenol-pyruvate
2.7.1.40
S-Acetyl-dihydrolipoamide-E
Ethanol
Pyruvate
1.2.1.3
2.3.1.12
1.2.4.1
1.2.4.1
1.1.1.1
6.2.1.1
Acetyl-CoA
2-Hydroxy-ethyl-ThPP
Acetate
Acetaldehyde
1.2.7.11
Glycolysis/gluconeogenesis pathway
Figure S3. Comparison of genes related to glycolysis and gluconeogenesis pathway in the genomes of Sphingomonas morindae sp. NBD5 and Sphingopyxis sp. USTB-05. The pink box in the figure indicate that the gene exists in Sphingomonas morindae sp. NBD5; the bright yellow box indicate that the gene exists in Sphingopyxis sp. USTB-05 and the orange box indicate that the gene exists in two strains.

## Slide 4
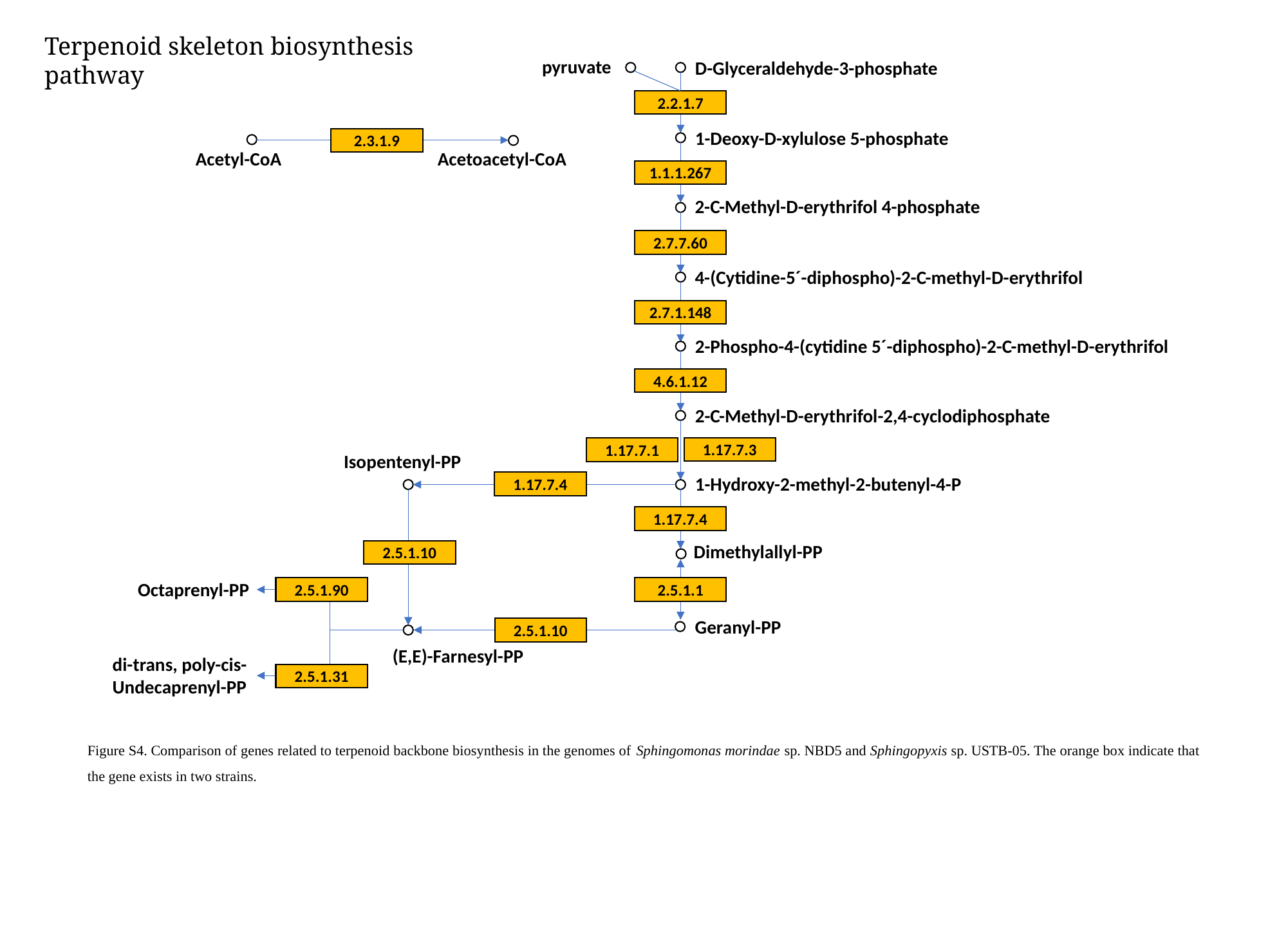

Terpenoid skeleton biosynthesis pathway
pyruvate
D-Glyceraldehyde-3-phosphate
2.2.1.7
1-Deoxy-D-xylulose 5-phosphate
2.3.1.9
Acetyl-CoA
Acetoacetyl-CoA
1.1.1.267
2-C-Methyl-D-erythrifol 4-phosphate
2.7.7.60
4-(Cytidine-5´-diphospho)-2-C-methyl-D-erythrifol
2.7.1.148
2-Phospho-4-(cytidine 5´-diphospho)-2-C-methyl-D-erythrifol
4.6.1.12
2-C-Methyl-D-erythrifol-2,4-cyclodiphosphate
1.17.7.1
1.17.7.3
Isopentenyl-PP
1-Hydroxy-2-methyl-2-butenyl-4-P
1.17.7.4
1.17.7.4
Dimethylallyl-PP
2.5.1.10
2.5.1.1
Octaprenyl-PP
2.5.1.90
Geranyl-PP
2.5.1.10
(E,E)-Farnesyl-PP
di-trans, poly-cis-Undecaprenyl-PP
2.5.1.31
Figure S4. Comparison of genes related to terpenoid backbone biosynthesis in the genomes of Sphingomonas morindae sp. NBD5 and Sphingopyxis sp. USTB-05. The orange box indicate that the gene exists in two strains.

## Slide 5
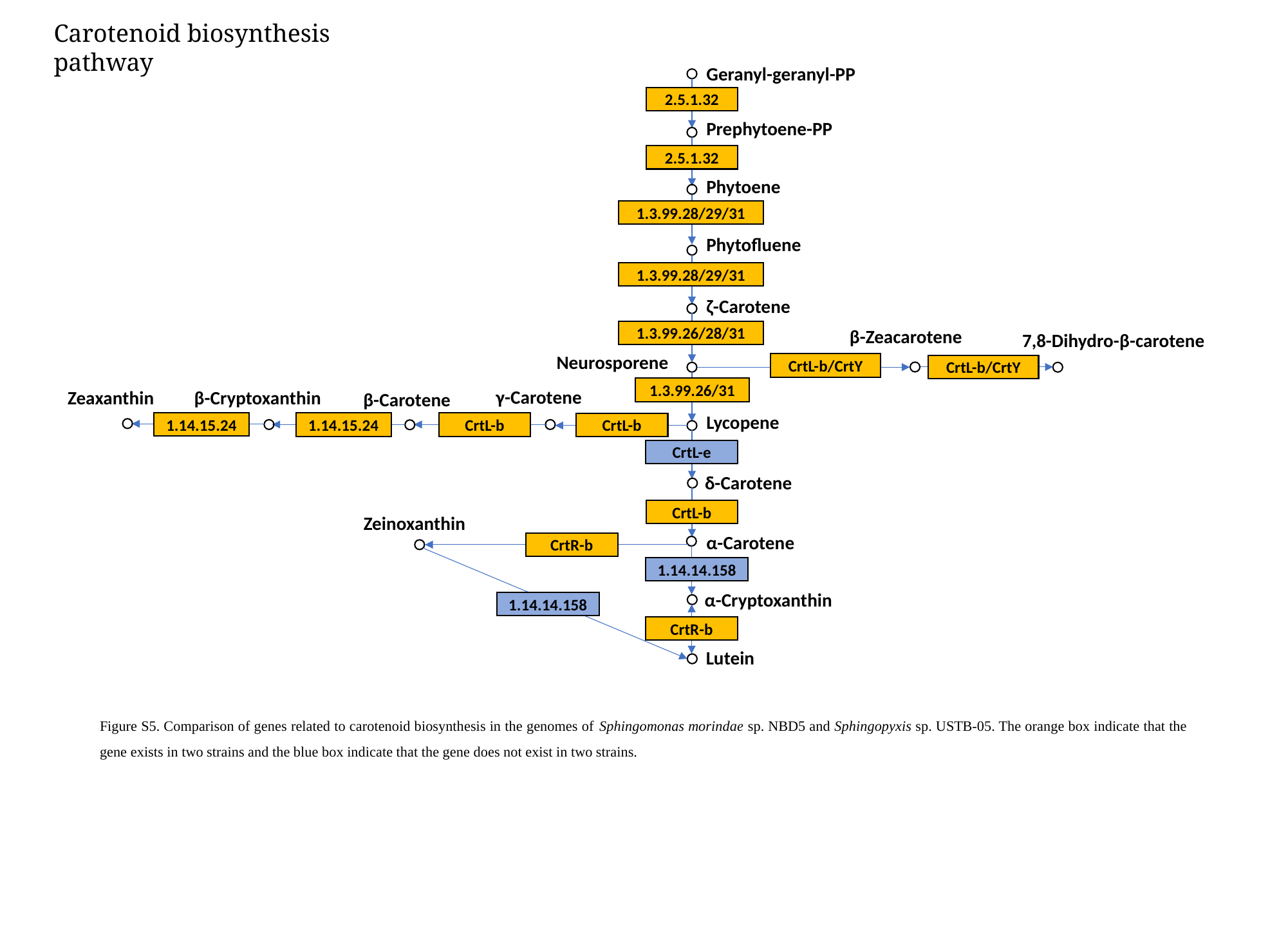

Carotenoid biosynthesis pathway
Geranyl-geranyl-PP
2.5.1.32
Prephytoene-PP
2.5.1.32
Phytoene
1.3.99.28/29/31
Phytofluene
1.3.99.28/29/31
ζ-Carotene
β-Zeacarotene
1.3.99.26/28/31
7,8-Dihydro-β-carotene
Neurosporene
CrtL-b/CrtY
CrtL-b/CrtY
1.3.99.26/31
γ-Carotene
Zeaxanthin
β-Cryptoxanthin
β-Carotene
Lycopene
1.14.15.24
1.14.15.24
CrtL-b
CrtL-b
CrtL-e
δ-Carotene
CrtL-b
Zeinoxanthin
α-Carotene
CrtR-b
1.14.14.158
α-Cryptoxanthin
1.14.14.158
CrtR-b
Lutein
Figure S5. Comparison of genes related to carotenoid biosynthesis in the genomes of Sphingomonas morindae sp. NBD5 and Sphingopyxis sp. USTB-05. The orange box indicate that the gene exists in two strains and the blue box indicate that the gene does not exist in two strains.
