# Supplementary material for: Comparative Genomic Analysis of Sphingomonas morindae sp. NBD5 and Sphingopyxis sp. USTB-05 for Producing Macular Pigment
Source: Microorganisms. 2023 Jan 19;11(2):266. doi: 10.3390/microorganisms11020266 (PMC9967899; doi:10.3390/microorganisms11020266)
Supplement: Supplementary file 1 [file microorganisms-11-00266-s001.zip › Figures S6-S8.pdf]

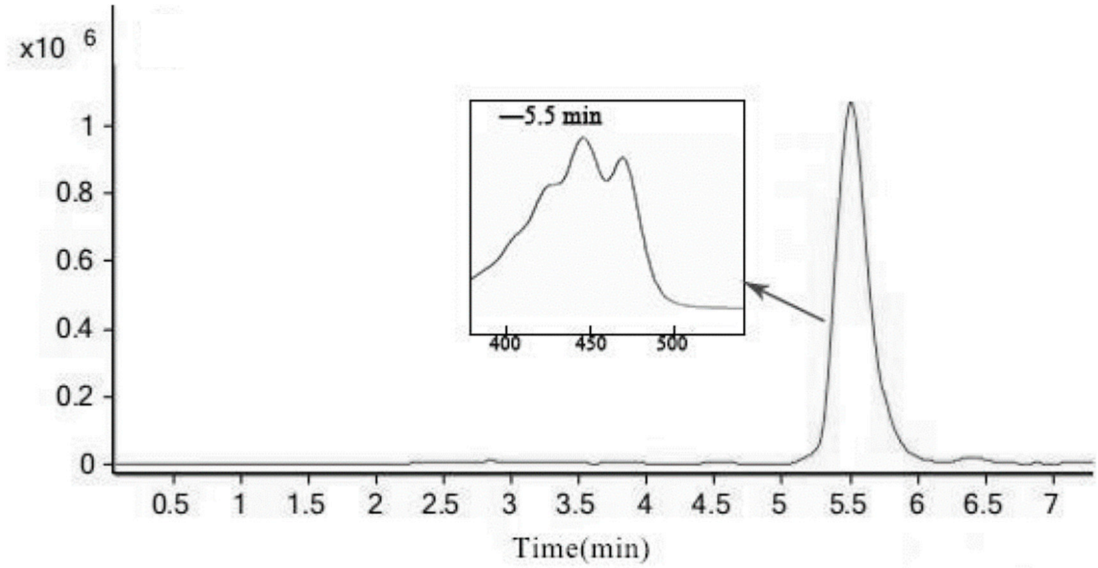

(**a**)

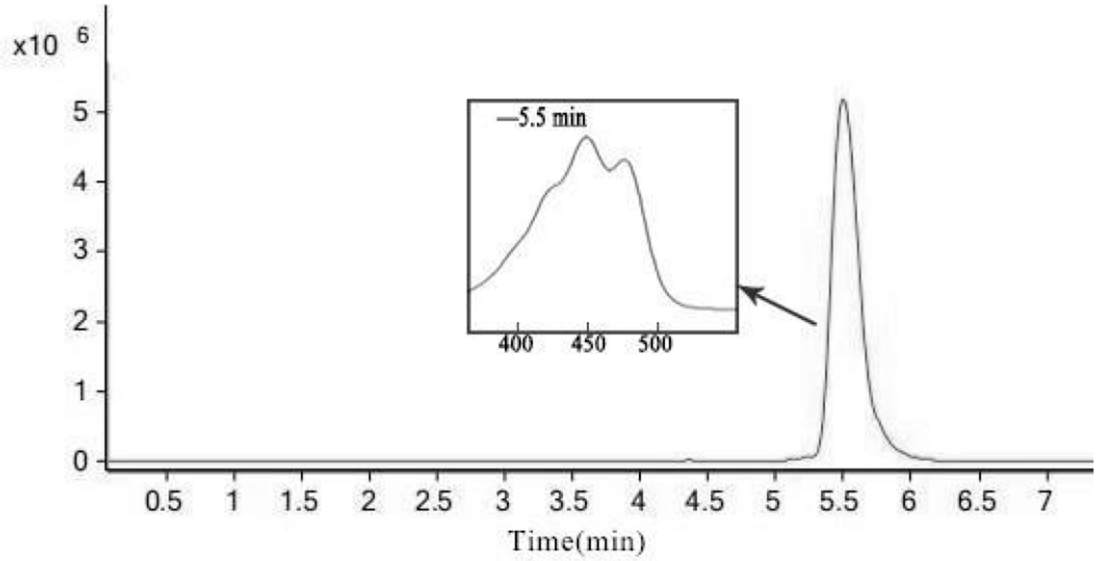

(**b**)

Figure S6. (**a**) Peaks and scanning profiles of standard lutein; (**b**) Peaks and scanning profiles of the extract of *S. morindae* NBD5 on LC-Q-TOF/MS [15].

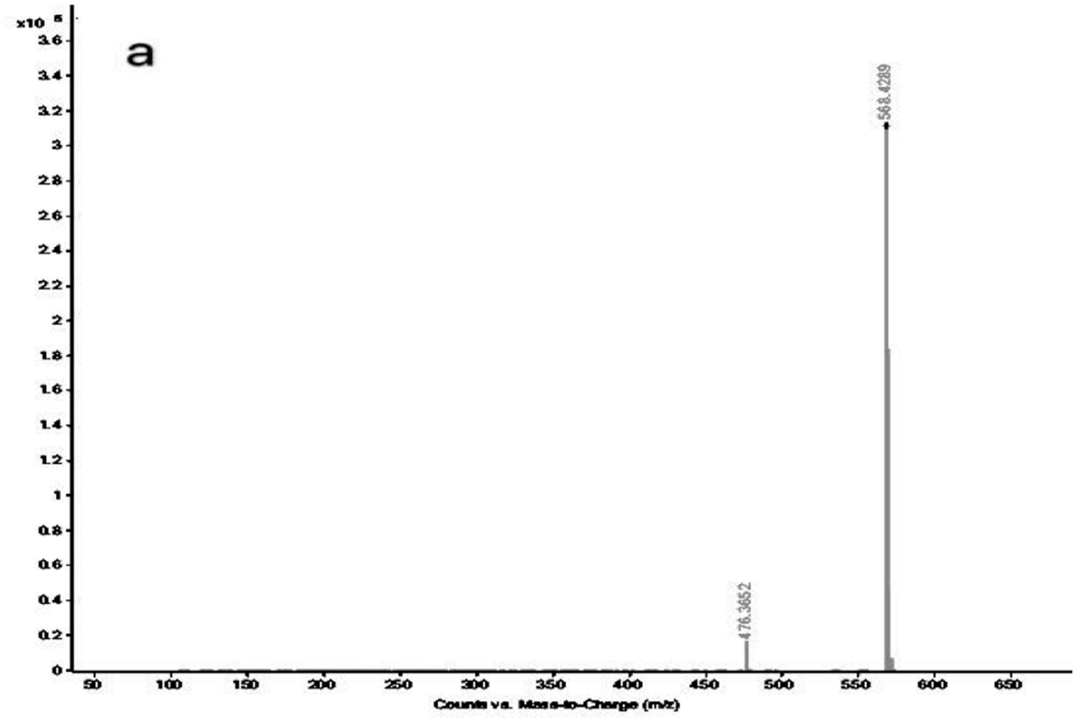

(**a**)

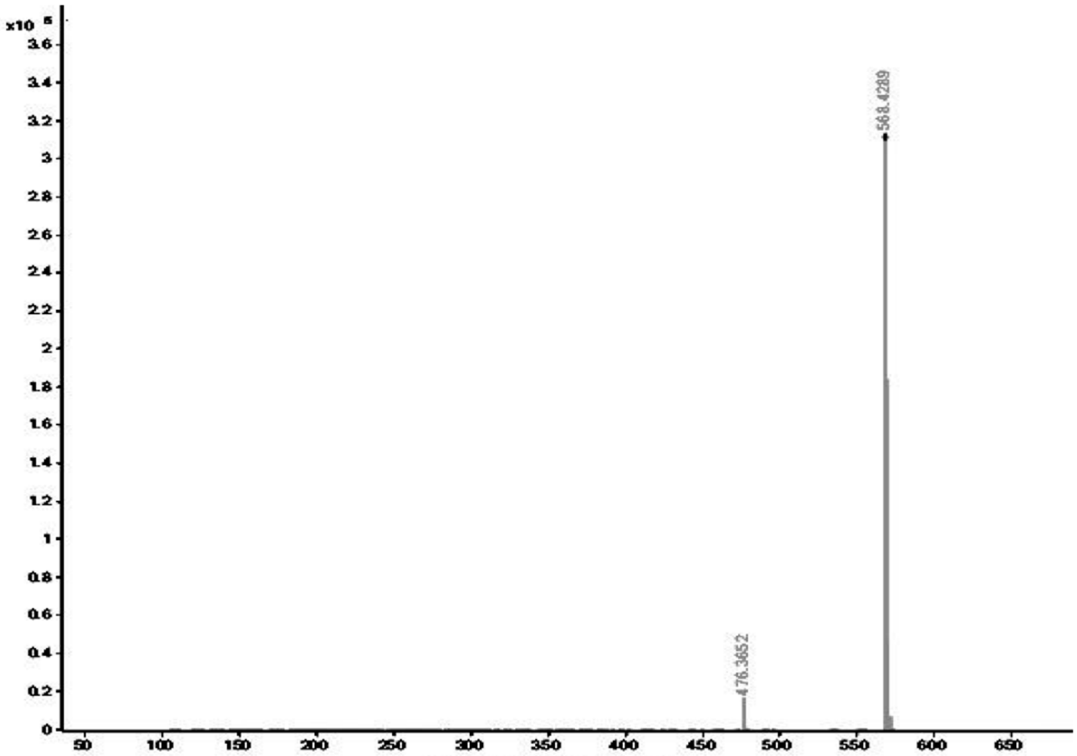

(**b**)

Figure S7. (**a**) First-order mass spectrometry of standard lutein on LC-Q-TOF/MS; (**b**) First-order mass spectrometry of the extract of *S. morindae* NBD5 on LC-Q-TOF/MS [15].

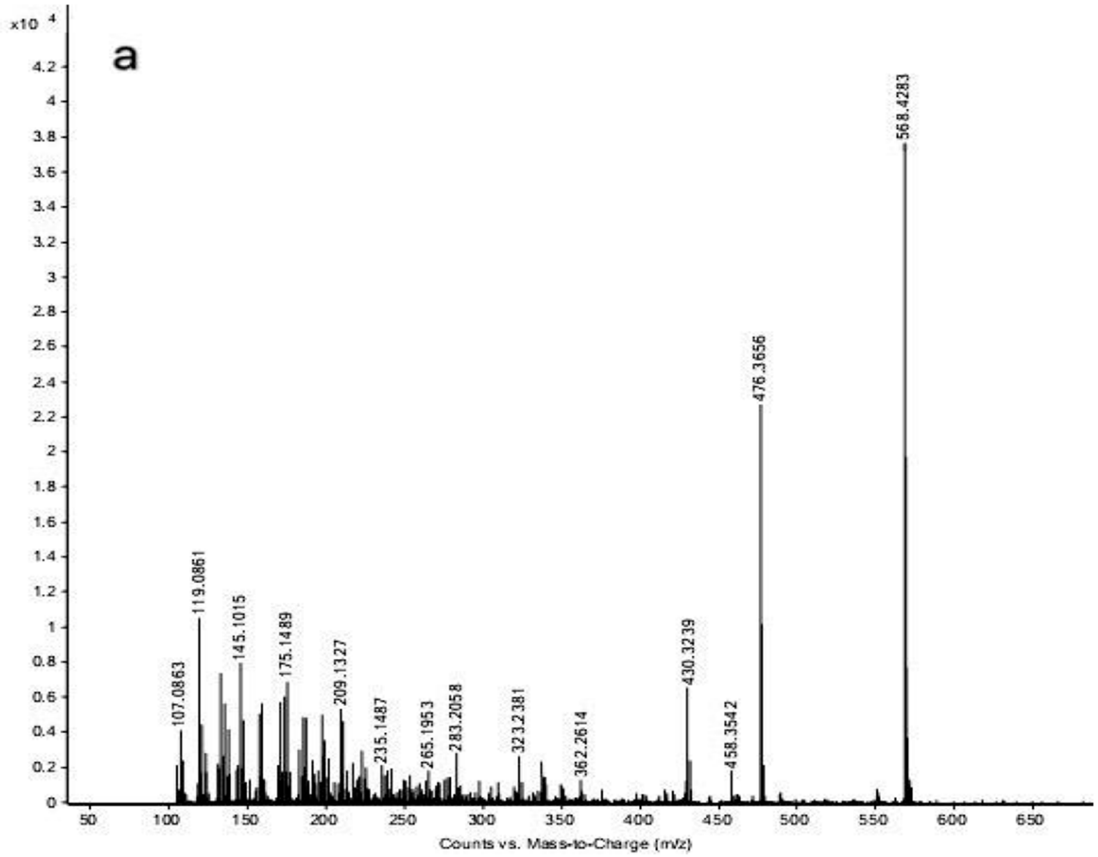

(**a**)

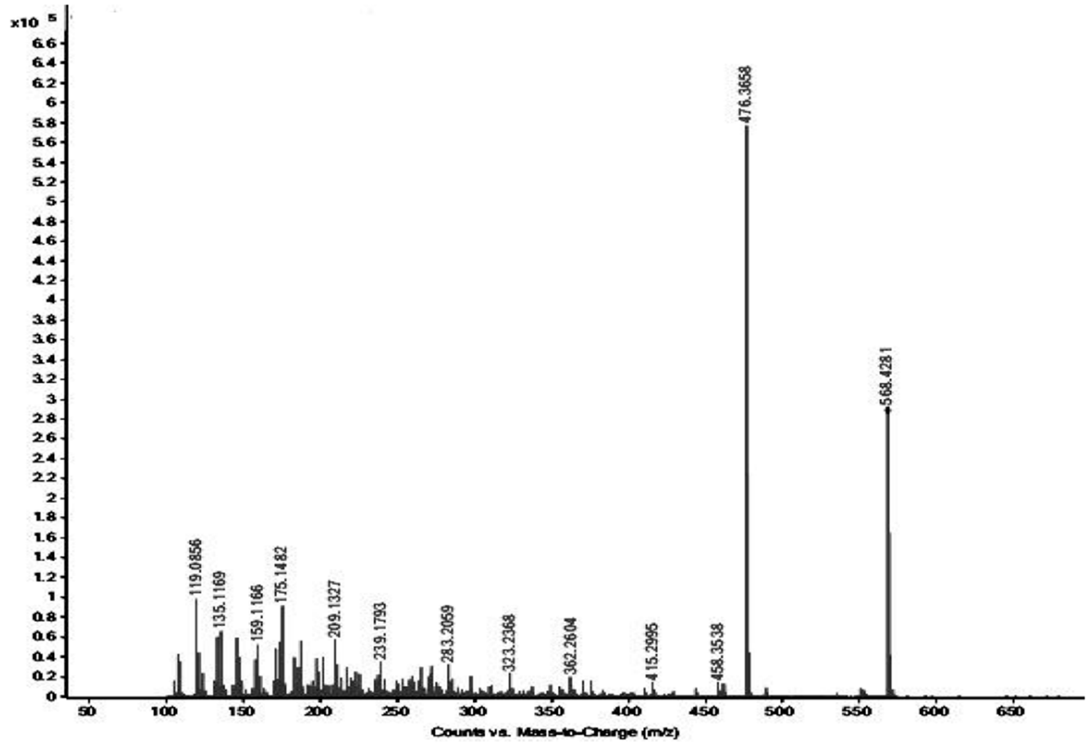

(**b**)

Figure S8. (**a**) Second-order mass spectrometry of standard lutein on LC-Q-TOF/MS; (**b**) Second-order mass spectrometry of the extract of *S. morindae* NBD5 on LC-Q-TOF/MS [15].

References

15. Wang, J.; Wu, P.; Chen, J.; Yan, H. Biodegradation of microcystin-RR by a new isolated *Sphingopyxis* sp. USTB-05. *Chin. J. Chem. Eng.* **2010**, *18*, 108–112.
